# Maternal Metformin Administration During the Pre-Gestation Period Improves Transient Cerebral Ischemia Injury in Male Offspring Rats

**DOI:** 10.34172/apb.43049

**Published:** 2024-09-15

**Authors:** Reyhaneh Vaali, Iraj Ahmadi, Fradin Sehati, Mina Ranjbaran, Marjan Nikbakhtzadeh, Fatemeh Nabavizadeh, Abbas Zareei, Ghorbangol Ashabi

**Affiliations:** ^1^Electrophysiology Research Center, Neuroscience Institute, Tehran University of Medical Sciences, Tehran, Iran.; ^2^Department of Physiology, School of Medicine, Tehran University of Medical Sciences, Tehran, Iran.; ^3^Department of Physiology, School of Medicine, Ilam University of Medical Sciences, Ilam, Iran.

**Keywords:** Metformin, Ischemia, Memory, Offspring, AMPK, Rats

## Abstract

**Purpose:**

It seems that maternal intervention, which may involve epigenetic mechanisms, can affect cerebral ischemia in offspring. Metformin consumption by the mother activates the AMP-activated protein kinase (AMPK) pathway. Metformin has also induced the AMPK and protected neurons in cerebral ischemia. This study investigates the effect of maternal metformin administration, which activates the AMPK pathway, on cerebral ischemia in offspring.

**Methods:**

Animals were separated into four groups: sham, 2-vessels occlusion (2VO), Met+2VO, Met+compound c (*CC*)+2VO. Female rats were administrated with metformin at a dose of 200 mg.kg^-1^ body weight for 2 weeks prior to mating. After the final metformin injection, each female rat was paired with an intact adult male to allow for mating. Sixty-days old offspring underwent cerebral ischemia and then memory-related tests were done.

**Results:**

Current data revealed that the neurological deficits score was reduced Met+2VO group (*P*<0.001), and the memory increased (*P*<0.001) in comparison to the 2VO. The Bcl-2/Bax ratio declined in the metformin group (*P*<0.001) while the brain-derived neurotropic factor (BDNF), c-fos, p-AMPK/AMPK ratio and Histone H3K9 acetylation in the hippocampus augmented significantly compared to the 2VO group (*P*<0.001).

**Conclusion:**

These findings indicated that the metformin intervention via AMPK activation could improve the movement disability, enhance spatial memory, increase neural plasticity, and augment the bioenergetics state and histone acetylation in the hippocampus of the offspring.

## Introduction

 Brain ischemia/reperfusion (I/R) is a pathophysiological condition with high mortality globally. It can be expressed when the organs or areas of body confront blood supply reduction, which is associated with reducing the transfer of oxygen and the nutrients to the tissues.^1^ Based on WHO statics, approximately 15 million people worldwide experience strokes, of which 5.5 million would annually die.^2^ Atherosclerosis, blood clots, vasoconstriction, congenital cardiac defects, embolism, head trauma, low blood pressure, sickle cell anemia, asphyxia, tachycardia, and some cancers are the conditions that cause this phenomenon.^3^ The reperfusion of cerebral blood flow after ischemia causes neuronal damage by the production, and invasion of ROS.^4,5^ Global cerebral ischemia reduces blood flow to hippocampus, striatum, and cerebral neocortex. These brain areas are involved in the expression of feelings, cognitions, emotions, and body movements of rodents.^6,7^ The hippocampus is susceptible to ischemia, and hypoxia.^8^ The function of hippocampal CA1 neurons is dominant for the formation, consolidation, and retrieval memory.^9^

 Recently, studies focused epigenetic mechanisms, and their intervention in the ischemic stroke pathogenesis, such as: DNA methylation, histone modifications (acetylation and methylation lysine residues in the terminal tail of histones H3, and H4) and RNA-based mechanisms.^10^ DNA methylation has a detrimental function in the start of neuronal cell death. It was suggested that treating cerebral ischemia by preventing methylation has therapeutic benefits.^11^ Besides, H3 and H4 acetylation reduction in the animal model of stroke causes severe brain damage.^10^

 5’-AMP-activated protein kinase (AMPK), a member of the serine/threonine (Ser/Thr) kinase family, is found in various cell types, such as skeletal muscles, and neurons.^12^ It is a cellular energy sensor. It catabolizes the body’s energy supply and removes the steps leading to ATP consumption, therefore responding to cerebral ischemia.^13^ The application confirmed the neuroprotective effects of AMPK activators on the neurological diseases, and dementia via metformin, resveratrol, and 5-aminoimidazole-4-carboxamide-1-β-d-ribofuranoside (AICAR).^14-16^ Metformin is an FDA-approved anti-diabetic drug for type-2 diabetes mellitus patient’s treatment.^17^ Metformin was shown to have an affirmative role to activate AMPK in Huntington’s disease, Alzheimer’s disease, and cerebral ischemia.^18^ Venna et al in 2012 reported that metformin-induced AMPK activation improved behavioral movements, and enhanced spatial memory in the 2-vessels occlusion (2VO) ischemia model.^19^ In stress conditions and lack of intracellular energy, metformin increases the expression of glucose transporter receptors on the cell surface and increases the entry of glucose into the cell.^20^ In ischemic rats, metformin administration lowered the percentage of brain edema, improved anxiety-like behavior, decreased the depressed-like behavior, enhanced working memory and spatial learning and memory, changed the content of superoxide dismutase and brain-derived neurotropic factor (BDNF).^1^

 Studies indicated that metformin administration in the prenatal period had a long-term effect on the metabolic phenotype, such as decreased the bodyweight of mice fetus, altered the glucose tolerance and increased the fasting glucose in high-fat diet offspring.^21^ Moreover, the activation of parental AMPK pathway has also been shown to alter specific genes expression, which involved acetylation and methylation of histones in offspring.^22,23^

 Here, we have examined the role of maternal metformin as an activator of AMPK pathway in the cerebral ischemia of offspring. In order to do this, intact male rats were mated with female rats who had received a 14-day metformin treatment. Male progeny rats who were two months old were given cerebral ischemia. Behavioral tests and molecular tests were assessed three days after induction of cerebral ischemia by 2VO model. Thus, an AMPK inhibitor, compound C (CC) was used to the exact role of AMPK activation by metformin in the transient cerebral ischemia model.

## Materials and Methods

###  Experimental animals 

 Ten Male and 10 female Wistar rats weighing 200 to 250 g were obtained from Institute Pasteur, Tehran, Iran. Animals were kept in standard cages under controlled temperature (22 ± 2 °C), humidity and a 12 hours light/ 12 hours dark cycle. All animals had freely access to food and water *ad libitum*. The acclimatization was done in order to being familiar with researcher and condition. The Experiments were permitted by the Ethics Committee of Tehran University of Medical Sciences, Tehran, Iran which is in accordance with international guidelines for animal experiments (Approval code: IR. TUMS.NI.REC.1399.047).

###  Mating protocol 

 Five female adult rats were orally treated with metformin at a dose of 200 mg/kg body weight, while another five female rats received oral saline treatment.^24^ Both treatments were administered daily for a period of fourteen days. Mating was initiated 24 hours after the last metformin or saline injection. Each female rat was then paired with an intact adult male for a duration of seventy-two hours, beginning the day after the final metformin/saline administration. The female pregnant rat was retained in a cage until parturition if the mating plug was seen. Male intact rats were used for other experiments in the lab. After parturition, pups and mothers were kept together for three weeks. After three weeks, male offspring were used for experiments. Induction of cerebral ischemia was done on adult male offspring (Sixty days old rats).

###  Study design

**Figure 1:**
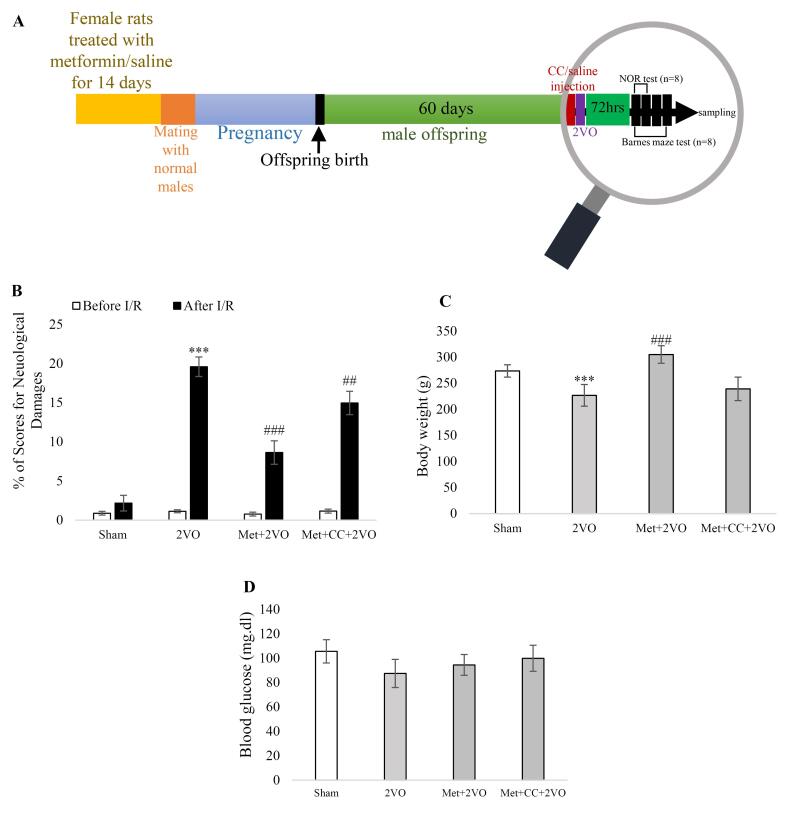
(A) The schematic design of the experiment. Rats were received the morphine pre-treatment for 14 days. Then, the mating was done on day 15. After two months, the offspring were injected with CC/saline 30 minutes before 2VO induction. Three days after occlusion, the behavioral tests such as neurological score, novel object recognition, and Barnes maze test were performed, and rats were sacrificed to assess the molecular factors.(B) The percentage of neurological deficits scores were detected before and after 2VO (n = 8). (C) Body weight was assessed in all groups of experiment and reported as gram unit (n = 8). (D) Blood glucose content was evaluated in all groups and reported in the shape of mg/dL (n = 8). Bars indicate the mean ± SEM. ***P < 0.001 versus the sham group, ##*P* < 0.01, ###*P* < 0.001 versus the 2VO group. Met, metformin (200 mg/kg); CC, compound c; 2VO, 2-vessel occlusion

 At first, male offspring rats were divided into four groups; (1) sham group; in this group, mothers of these rats’ received saline for 14 days, and then offspring were anesthetized, and after a ventral midline incision in the neck region, both common carotid arteries (CCAs) was exposed without any occlusion. (2) 2VO group; in this group, mothers of these rats received saline for 14 days, then offspring were anesthetized, and after a ventral midline incision in the neck region, both CCAs were exposed and occluded for 30 minutes. (3) metformin (met) + 2VO group; in this group, mothers of these rats received metformin (200 mg.kg^-1^) for 14 days, and then offspring were anesthetized, and after a ventral midline incision in the neck region, both CCA was exposed and occluded for 30 minutes. (4) met + CC + 2VO group; in this group, mothers of these rats received metformin (200 mg.kg^-1^) for 14 days, and then offspring were anesthetized, and 30 minutes prior to 2VO surgery, rats received CC (5 μg/μL by intravenous (i.v) injection) and then both CCA was exposed and occluded for 30 minutes (Figure 1A). A total of 34 offspring rats were utilized in the study.

###  Surgical procedure of transient CCA ligation

 Offspring rats were anesthetized with chloral hydrate (400 mg/kg, i.p.) (Sigma Aldrich Co. USA), and their body temperature was monitored using a probe and maintained at 37 °C with a heating pad. A ventral midline incision was made in the neck region to expose the left and right CCAs, which were carefully separated from the adjacent vagus nerves. Both CCAs were occluded using aneurysm clips for 30 minutes to induce transient cerebral ischemia, a method commonly used in studies of cerebral blood flow and ischemic injury. After the occlusion period, the clips were removed to allow for reperfusion. This procedure effectively mimics the conditions of I/R, allowing for the assessment of subsequent neurological outcomes. Importantly, there was no significant mortality rate among the experimental groups, and the animals were allowed to recover from anesthesia before being returned to their cages. This approach is consistent with established protocols in the field, ensuring reproducibility and reliability of the results.^25,26^

###  Body weight and blood glucose assay

 The digital balance measured body weight four days after the induction of I/R. A glucometer (TaiDoc Technology Corporation, Taiwan) assessed blood glucose levels four days after the induction of I/R.^18^

###  Neurological deficit evaluation

 Neurological outcomes were evaluated using standard neurological score parameters (a maximum score of 25).^27^ Neurological tests were done 96 hours (4 days) after induction of I/R (n = 8). In brief, neurological scores were applied to the six sensory-motor activities as follows: hair roughed up, decreasing movement or brady-pragia, enhancement of response to ear-palpating and eyelid ptosis considered maximum 1 score each. Up-warping head or hunched posture, eye misclosure or patency, circling behavior, splayed-out hind limb and hyperspasmia considered maximum 3 scores each and Myasthenia of limbs considered maximum 6 scores.^27^

###  Novel object recognition test (NOR)

 All behavioral tests were done from 10:00 am to 02:00 pm. NOR were evaluated on 4, 5 and 6 days after induction of 2VO (n = 8). NOR test is done on an open field apparatus (50 × 50 × 40 cm). On the first day (4 days after I/R), the habitation is done for 20 minutes. On the second day (5 days after I/R), rats were left on open field apparatus with two equal items for 6 minutes. On the third day (6 days after I/R), one of two equal items was changed with a different item (the new one is different in shape, color, and texture); after changing one of the items with the new one, rats were left in the open field apparatus for 6 minutes. The total time spent near each item was recorded by chronometer and by a blind person. Exploring is considered nosing and sniffing around items when rats are near them (less than 2 cm). The percentage of recognition index (RI) is reported by using the following formula: [time spent in exploring the novel item /time spent in exploring both items] × 100.^28^

###  Barnes maze test

 Barnes maze was completed 4-7 days after induction of 2VO (n = 8). The Barnes maze is a 122-cm-diameter circular platform on a 1-meter stand with 18 holes (each hole has 10 cm diameter and is around the circumference of the platform). In addition, a black box named as escape box (20 × 15 × 12 cm) was embedded under one of the holes. Four visual cues were placed surrounding dark walls. A light power (500 W, 1000 lx) was positioned on the maze surface. A day before starting the learning process, rats were kept on an escape box for 2 minutes for habitation. On the first day until the third day (4-6 days after I/R), training and acquisition trials were done, and rats learned the position of the escape box. Time to find the escape box (latency time) and the number of errors on all three days were recorded by a blinded investigator. On day four (7 days after I/R), the escape box was removed, and the time to enter the target hole and the number of errors during 90 seconds were recorded.^29^

###  Sacrifice and tissue preparation

 On day seven, following the behavioral tests, the rats were deeply anesthetized using ketamine hydrochloride (50 mg/kg) and xylazine (4 mg/kg) via intraperitoneal injection. Each experimental group (n = 8) was divided into two parts. In the first part (n = 4), the animals were perfused through transcranial injection with phosphate-buffered saline (PBS, pH 7.4), followed by perfusion with 4% paraformaldehyde in 0.1 M PBS (pH 7.4). Subsequently, the brains were removed and fixed in 4% paraformaldehyde for four days. The fixed brains were then embedded in paraffin using a specialized tissue processor.

 The hippocampi from the remaining four rats in each group were utilized for Western blotting and enzyme-linked immunosorbent assay (ELISA). These hippocampi were collected on ice, frozen in liquid nitrogen, and stored at −80 °C (n = 4). Total protein from the hippocampus was lysed and extracted following the protocol established by Niimura et al.^30^

###  H & E staining

 Animals in each group were chosen randomly to ensure unbiased representation for tissue preparation. Coronal sections (4–5μm) of hippocampal formation were prepared for H&E staining. After sectioning paraffin-embedded brains, the slides were deparaffinized with descending xylene and ethylene glycol and embedded in H&E stains. Slides were put into ascending xylene and ethylene glycol and covered by specific adhesive glue into lamella. Neurons were calculated when a visible nucleolus was detected within the counting frame came into focus. Cells with unclear nuclei were not counted. The mean percentage of dead neurons was reported by counting the damaged neurons divided by the total number of neurons of the CA1 region. An optical microscope did cell count at a magnification of × 400 (n = 4).^31^

###  Protein’s extraction and western blotting

 Protein extracted from the supernatant was assayed using the Bradford method.^32^ Sixty micrograms protein were loaded on SDS-PAGE on 12% polyacrylamide gel (n = 4). Lanes on each gel were also loaded with pre-stained protein markers to evaluate finalizing of electrophoretic transfer time. After transfer electrophoretic gel to polyvinylidene fluoride membrane, the blots were kept in a blocking buffer (5% nonfat dry milk in Tris-buffered saline containing 0.05% Tween-20 (TBS-T)) for 1 hour at room temperature, and then incubated 18 hours at 4 °C with primary antibodies against these factors: Bax, Bcl-2, pAMPK, total AMPK, histone H3 and acetyle-H3K9 (1/1000, Cell Signaling Technology Co. USA) antibodies. The blots were then stripped for antibodies removal by incubation for 15 min with restored Western blot stripping buffer at room temperature, then re-blocked and incubated for 1 hour at room temperature. Afterward, the β-actin antibody ((1/1000, Cell Signaling Technology Co. USA) was incubated for 3 hours and after washing with TBS-T, blots were incubated with the appropriate secondary antibody for 1.5 hours. Antigens were visualized and exposed to radiography film. The densitometric scan of films quantified the result, and analysis was done by ImageJ, measuring the integrated density of protein bands after background deletion. β-actin was applied as a loading control.^30^

###  ELISA technique

 Specific lysis buffer was added to lyse the hippocampi tissues and lysate underwent centrifuging at 15 000 rpm for 5 minutes at 4 °C, and the protein concentration of supernatants was calculated by the Bradford dye method.^32^ BDNF and c-fos ELISA assay kits (Abcam Co. USA) were applied by conforming to the manufacture guideline (n = 4). BDNF and c-fos concentrations were measured by postulating the absorbance criteria at 405 nm and reading the light absorbance by spectrophotometry.

###  Statistical analysis

 A power analysis for behavioral tests was conducted prior to the experiments, using an alpha level of 0.05 and a desired power of 0.80, which indicated that a minimum sample size of 8 per group was necessary to reliably detect the expected effect sizes. Therefore, number of animals for behavioral tests were 8 in each group. Number of animals for molecular tests and histological assessments were 4 in each group. Molecular experiments were repeated three times, technically. Mean ± SEM (standard error of the mean) was used to express the data, which was processed using Graph Pad Prism® 5.0. One-way analysis of variance (ANOVA) followed by post hoc analysis (Tukey and Newman–Keuls test) were used to determine statistical significance. P-values less than 0.05 (*P* < 0.05) were considered statistically significant and 95% confidence intervals were calculated to provide a range of values within experimental groups.

## Results

###  Mortality rate in experimental groups 

 In this study, a total of 32 offspring rats were utilized across all experimental groups. During the course of the experiment, two rats from the 2VO group unfortunately died. To maintain consistency in group size, these two rats were replaced, ensuring that each group continued to consist of eight rats. This results in a mortality rate of 6.25% for the 2VO group.

###  Maternal metformin improves neurological scores and non-spatial memory in the I/R induced offspring

 The changes in neurological deficit scores are demonstrated in Figure 1B. After the induction of I/R, rats in the 2VO group exhibited elevated neurological scores compared to the sham group (*P* < 0.001). Additionally, the neurological deficit scores were significantly reduced in the Met + 2VO group compared to the 2VO group after I/R (*P* < 0.001). However, the CC treatment increased the neurological deficit scores in the Met + CC + 2VO group compared to the 2VO group after I/R (*P* < 0.01).

###  Metformin treatment of the mother could alter the body weight in 2VO rat model offspring

 The body weight decreased in the 2VO group compared to the sham group (*P* < 0.001). In the Met + 2VO group, the body weight increased significantly compared to the 2VO group (*P* < 0.001, Figure 1C). According to Figure 1D, the blood glucose content did not show any changes in any of the groups (*P* > 0.05).

###  Maternal metformin treatment significantly reduced learning and memory deficits which 2VO induces in offspring

**Figure 2:**
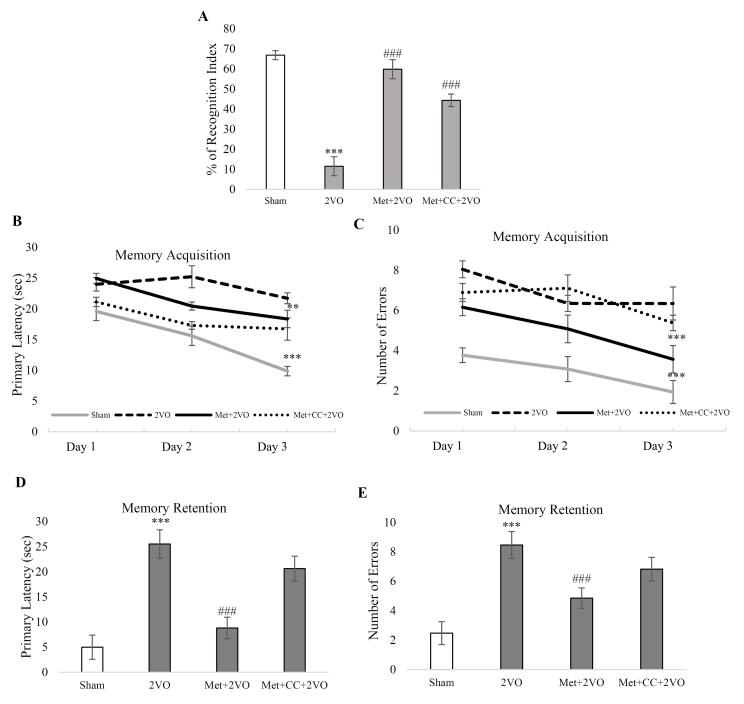
(A) Percentage of recognition index was measured by NOR test (n = 8). (B) The Barnes maze test measured memory acquisition latency. (C) The Barnes maze test reported the number of errors in memory acquisition. The Barnes maze test assessed. (D) Memory retention latency. (E) Memory retention errors were measured by the Barnes maze test (n = 8). Data were presented as Mean ± SEM. ***P < 0.001 vs. the sham group, ###*P* < 0.001 vs. the 2VO group. Met, metformin (200 mg/kg); CC, compound c; 2VO, 2-vessel occlusion

Figure 2A depicts that the change in the object recognition index was significantly decreased in the 2VO group compared to the sham group (*P* < 0.001). The percentage of recognition index enhanced in the Met + 2VO group compared to the 2VO group (*P* < 0.001). Object recognition index in Met + *CC* + 2VO group significantly increased compared to 2VO group (*P*< 0.001).

 As shown in Figure 2B, the 2VO animals required more time to seek out the black escape box than sham rats (*P* < 0.001). In contrast, the Met + 2VO group had considerably shorter escape latencies to the escape box during the final training sessions than the sham group (*P* < 0.001). Also, the Met + 2VO group made fewer errors in memory acquisition than the sham group (*P* < 0.001, Figure 2C). In addition, the memory retention latency and the number of errors increased in the 2VO group compared to the sham group (*P* < 0.001). In Met + 2VO, the memory retention latency and the number of errors decreased in comparison to the 2VO group (*P* < 0.001, Figures 2D and 2E). The *CC* application did not have any significant effect in comparison to the 2VO group on Barnes maze indices.

###  Metformin pretreatment by mothers protects neurons against cell death in the 2VO rat model offspring

**Figure 3:**
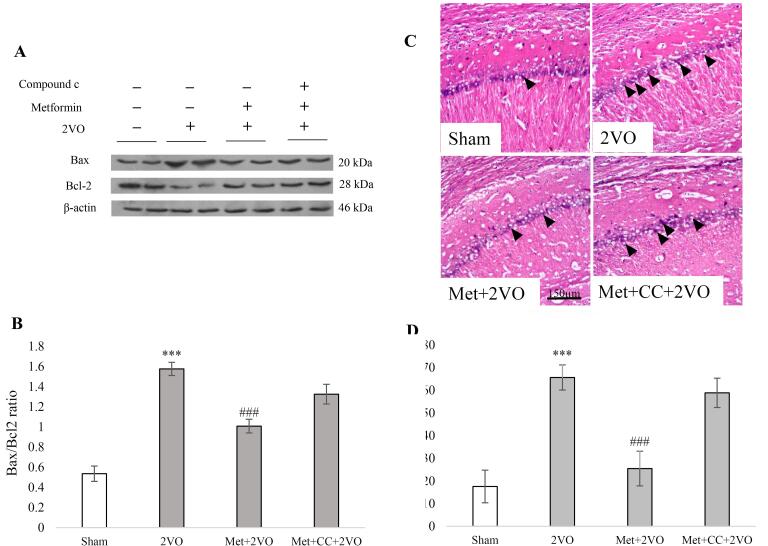
(A) Western blot analysis for assaying the effect of metformin pretreatment on the Bax and Bcl-2 levels in the hippocampus. (B) The effect of metformin on Bax/Bcl-2 ratio. The density of Bax and Bcl-2 were measured, and the ratio was calculated. (C) Percentage of apoptosis in CA1 hippocampus cell. The black line indicates a scale of 150 microns Data were presented as Mean ± SEM. ***P < 0.001 vs. the sham group, ###*P* < 0.001 vs. the 2VO group. Met, metformin (200 mg/kg); CC, compound c; 2VO, 2-vessel occlusion

 The levels of the anti-apoptotic Bcl-2 and pro-apoptotic Bax were studied to examine apoptotic signaling. Figures 3A and 3B illustrate the representative blots of Bax and Bcl-2, as well as the ratio of Bax/Bcl-2. Compared to the sham group, the graph shows a substantial increase in the Bax/Bcl-2 ratio in the 2VO group (*P* < 0.001). There is a significant reduction in the Bax/Bcl-2 ratio in the Met + 2VO group compared to the 2VO group (*P* < 0.001). The number of dead cells in the Met + 2VO group was lower than in the 2VO group (*P* < 0.001, Figures 3C and 3D), but the number of dead cells in the 2VO group increased significantly compared to the sham group (*P* < 0.001). CC treatment could not alter the effect of maternal metformin treatment on neuronal survival significantly compared to the 2VO group (*P* > 0.05).

###  Maternal metformin regulates hippocampal phosphorylated AMPK and Histone H3K9 acetylation levels in 2VO rat model offspring

**Figure 4:**
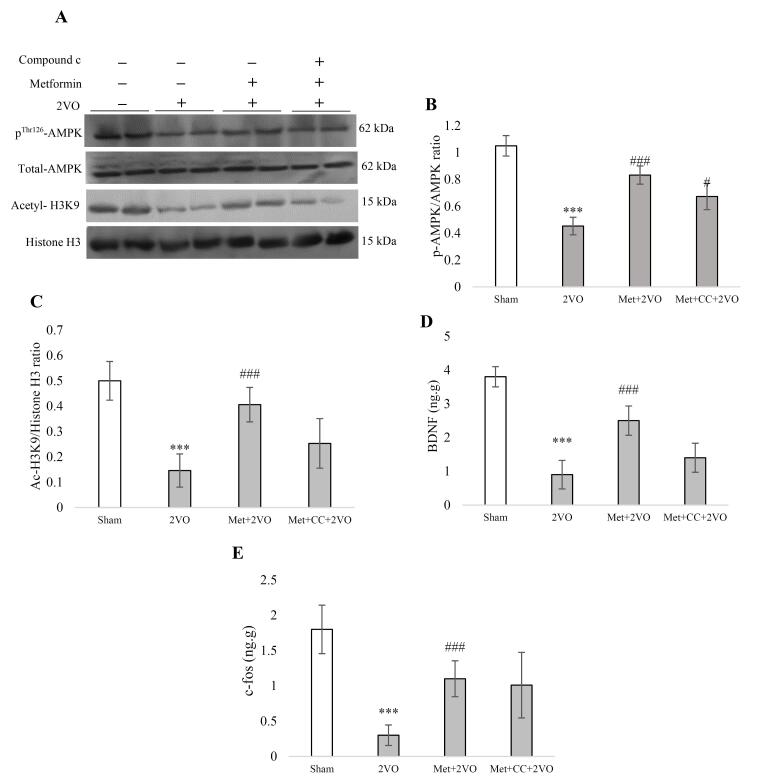
(A) Western blot analysis to measure the effect of metformin pretreatment on the pAMPK/AMPK and acetyl H3K9/histone H3 ratio. (B) The density of phosphorylated AMPK band to total AMPK bands was measured, and the ratio was calculated. (C) The density of acetyl H3K9 band to the total histone H3 bands was assayed and the ratio was calculated. (D) ELISA assay kit was performed for assaying the BDNF content and expressed as a ng. g-1. (E) ELISA assay kit was performed to measure the c-fos content and expressed as an ng/g. Data were presented as Mean ± SEM. ***P < 0.001 vs. the sham group, #*P* < 0.05, ###*P* < 0.001 vs. the 2VO group. Met, metformin (200 mg/kg); CC, compound c; 2VO, 2-vessel occlusion

 We assessed total AMPK and pAMPK to calculate the pAMPK/AMPK ratio in Figures 4A and 4B. I/R reduced the pAMPK/AMPK ratio compared to the sham group (*P* < 0.001). Also, a significant increment of maternal metformin treatment on pAMPK/AMPK ratio in the Met + 2VO group compared to the 2VO group was found (*P* < 0.001). The AMPK Inhibitor (*CC*) reduced this ratio significantly compared to the Met + 2VO group (*P* < 0.05).

 Analysis of the Western blots showed that in the 2VO group acetyl-H3K9 significantly reduced compared to the sham group (*P* < 0.001, Figure 4A and 4C). The acetylation of histone H3K9 increased following metformin treatment in the Met + 2VO group compared to the 2VO (*P* < 0.001).

###  Maternal metformin treatment enhanced hippocampus BDNF and c- fos levels in the 2VO rat model offspring

 As shown in Figure 4D, the level of BDNF was significantly reduced in 2VO rats’ hippocampus compared to the sham group (*P* < 0.001). Significant elevations were found in BDNF in the Met + 2VO group compared to the 2VO group (*P* < 0.001). Figure 4E reports that the c-fos protein level decreased in the 2VO group compared to the sham group (*P* < 0.001). The c-fos level increased in metformin-treated rats compared to the 2VO group (*P* < 0.001).

## Discussion

 Maternal administration of metformin (200 mg.kg^-1^) significantly improves memory deficits, reduces apoptosis, and enhances neural plasticity factors such as BDNF and c-fos protein levels. It also induces AMPK phosphorylation in male offspring and increases H3K9 acetylation. However, adding *CC* before the induction of 2VO in offspring reduces AMPK phosphorylation and inhibits the protective effects of maternal metformin treatment against I/R injury.

 Metformin is used for known disorders such as polycystic ovary syndrome, obesity, and diabetes. The metformin has a continuum of effects, which depend on several causes in ischemia.^33^ The metformin’s preservative role to activate AMPK in cardiac, neuronal, and renal cells under the ischemia model was approved by previous studies.^24^ Metformin activates the AMPK pathway in cerebral ischemia through several mechanisms. Primarily, it inhibits mitochondrial complex I, which increases the AMP/ATP ratio and directly activates AMPK, enhancing cellular energy metabolism and promoting neuroprotection during ischemic events. Additionally, metformin may modulate gut microbiota and gastrointestinal signaling, further influencing AMPK activation.

 Key downstream targets of AMPK relevant to neuroprotection include acetyl-CoA carboxylase (ACC), which regulates fatty acid metabolism, and the CREB, crucial for expressing neurotrophic factors like BDNF. By phosphorylating these targets, AMPK activation supports neuronal survival and enhances synaptic plasticity, ultimately improving cognitive function and neurological outcomes following ischemic injury.

 Based on these findings, the question is: does maternal metformin treatment have a significant effect on offspring’s neuronal function, especially in the ischemic model? Metformin treatment during pregnancy has some effects on offspring’s gonadal function.^34^ Shreds of evidence showed that maternal metformin treatment during pregnancy could improve memory and hippocampal insulin-like growth factor 2 in offspring.^35^ Moreover, metformin treatment during gestation alters myogenesis and mitochondrial biogenesis of skeletal muscle via the activation of AMPK signaling in offspring.^36^ Some data showed that prescribing metformin before gestation has no role on abortion or delivery rate.^37,38^ Therefore, the current study showed that metformin treatment before gestation has effective roles on offspring cerebral ischemia, probably by AMPK activation.

 The potential of metformin against cerebral ischemia is well-established. It was reported that metformin could improve sensory-motor function, learning and memory in global and focal cerebral ischemia.^1,39^ Previous data showed that metformin could improve cerebral ischemia via AMPK signaling pathway.^18^ According to available research, treating ischemic offspring with metformin may improve their sensory-motor and spatial/non-spatial memory functions. Metformin has been shown to activate AMPK in offspring, and this can lead to AMPK signaling, which in turn can induce neuronal plasticity and memory-related factors like BDNF and CREB.^24,40^

 Metformin is used to control body weight, but our data showed that metformin pretreatment of mothers increased ischemic offspring’s body weight. These changes in body weight might be related to their locomotion and searching for food. Although, many studies revealed that the body weight and blood glucose had no significant changes in the cerebral ischemia.^18,41^ In another study, it was recommended that metformin treatment for 14 days doesn’t affect blood glucose in focal ischemic rats, which doesn’t reveal any sign of diabetes.^42^

 It was shown that under oxidative stress and neurodegeneration, metformin operates by phosphorylating AMPK, which in turn stimulates downstream signaling pathways in neurons.^43^ Ge et al showed that the number of live neurons in the hippocampus increased when metformin was administered after acute global brain ischemia.^39^ The current results agree with previous data and our behavioral studies that AMPK activation by metformin pretreatment in pre-gestational period could reduce neuronal cell death during I/R.

 Data showed that metformin treatment just before gestation increased AMPK in offspring and AMPK activation is responsible for the beneficial role of metformin pre-gestational treatment. The metabolo-epigenetic outlook of metformin suggests that it can alter DNA and histone modification.^44^ Metformin treatment alters histone modifications; for example, metformin reduces H3K4 or H3K27 methylation in cancer cells.^45,46^ However, Fang and colleagues reported that metformin could induce neuronal viability by enhancement of total H3 acetylation,^47^ but Sunagawa et al disclosed that metformin protects myocardial cells by reduction of histone H3K9 acetylation in myocardial cells.^48^ Research has shown that histone acetylation can improve neurogenesis and increase the capacity for regeneration in brain injury in the middle cerebral artery occlusion model.^49^ The participation of histone acetylation in the acute phase of stroke is well documented by other experiments.^50-52^ Another point of view expressed that histone acetylation treatment can be used in post-stroke recovery and vascular remodeling.^49^ Meng et al presented that the effect of metformin was associated with the intervention in the cell cycle, as determined by diminishing the phosphorylated histone-3 content.^53^ According to the present findings, it may be suggested that administering metformin before pregnancy elevated AMPK levels in the mother, which in turn boosted H3K9 acetylation in the child.^54^

 The findings of this study suggest several important future research directions and clinical implications. Investigating the long-term effects of maternal metformin treatment on offspring cognitive outcomes and the role of AMPK activation in mediating these benefits could provide valuable insights. Additionally, examining the relationship between H3K9 acetylation and memory-related gene expression, as well as assessing the potential of metformin as a preventive strategy for neurodegenerative disorders, may enhance our understanding of its therapeutic effects. Optimizing treatment timing and dosage, exploring sex-specific differences, and translating these findings to human studies through clinical trials could lead to evidence-based guidelines for metformin use during pregnancy, ultimately promoting long-lasting neuroprotective effects in offspring.

 As a limitation of the study, the levels of the specified proteins were not assessed before the induction of I/R injury. Measuring the baseline protein levels prior to I/R could have provided additional insights into the effects of maternal metformin administration on the offspring’s cerebral ischemia outcomes. Despite this limitation, our findings suggest that H3K9 acetylation might alleviate cerebral ischemia by enhancing neuronal protective factors, such as anti-apoptotic proteins, BDNF, and c-fos levels. Furthermore, studies have indicated that H3K9 acetylation may ameliorate memory deficits through the upregulation of BDNF, c-fos, and CREB expressions. These insights underscore the potential mechanisms through which maternal metformin administration may confer neuroprotective effects in offspring.^55,56^

## Conclusion

 While numerous studies have investigated the effects of metformin on cerebral ischemia, there is a notable paucity of research focusing on the role of epigenetic mechanisms in this context, particularly in relation to neurodevelopment, learning, and memory. Key factors such as pAMPK, ATP cellular consumption, histone acetylation, and DNA methylation-epigenetic mechanisms that merit further exploration-remain inadequately addressed. This study demonstrates that maternal administration of metformin prior to gestation activates AMPK in offspring, likely enhancing cognitive functions through the induction of H3K9 acetylation and the promotion of neuronal plasticity. Although the findings suggest that metformin improves neurological outcomes via AMPK activation, the precise molecular mechanisms involved are not yet fully understood. Potential pathways include increased glucose uptake, enhanced insulin sensitivity, and the inhibition of “mechanistic target of rapamycin complex 1” signaling, all of which may contribute to neuroplasticity. Furthermore, maternal metformin treatment may upregulate memory-related genes such as BDNF, c-fos, and CREB through H3K9 acetylation, thereby facilitating improved cognitive function. Comprehensive research is necessary to elucidate these pathways and their specific roles in mediating the observed benefits, thereby positioning metformin as a promising therapeutic agent for epigenetic interventions in future generations.

## Competing Interests

 The authors declare they have no conflict of interests.

## Ethical Approval

 The study protocol was reviewed and approved by the ethical committee of the Faculty of Medicine, Tehran University of Medical Sciences.
